# A Comparative Study on Two Types of Porcine Acellular Dermal Matrix Sponges Prepared by Thermal Crosslinking and Thermal-Glutaraldehyde Crosslinking Matrix Microparticles

**DOI:** 10.3389/fbioe.2022.938798

**Published:** 2022-08-05

**Authors:** Xing Huang, Yi Ding, Wenqian Pan, Lin Lu, Rui Jin, Xiao Liang, Mengling Chang, Yinmin Wang, Xusong Luo

**Affiliations:** ^1^ Department of Plastic and Reconstructive Surgery, Shanghai 9th People’s Hospital, Shanghai Jiao Tong University School of Medicine, Shanghai, China; ^2^ Shanghai Key Lab of Tissue Engineering, Shanghai 9th People’s Hospital, Shanghai Jiao Tong University School of Medicine, Shanghai, China; ^3^ Jiangsu Unitrump Biomedical Technology Co.,Ltd., Jiangsu, China

**Keywords:** porcine dermal matrix, microparticles, sponge, crosslinking, integration

## Abstract

Common commercial porcine acellular dermal matrix (PADM) products take the form of a thin membrane. Given its dense structure, delaying vascularization after implantation remains an issue to be solved. In addition, overlaying multiple sheets to address deep wounds and large tissue defects that are difficult to repair by self-tissues could hinder tissue ingrowth, angiogenesis, and integration. Here, we creatively prepared PADM microparticles through a homogenizing treatment and crosslinked them to ADM sponges by thermal crosslinking (VT-ADM) and thermal-glutaraldehyde crosslinking (GA-ADM). The resulting VT-ADM was thicker than GA-ADM, and both maintained the natural dermal matrix microstructure and thermal stability. The porosity of GA-ADM (mean 82%) was lower than that of VT-ADM (mean 90.2%), but the mechanical strength and hydrophilicity were significantly higher. The two types of ADM sponges showed no obvious difference in cell adhesion and proliferation without cytotoxicity. Furthermore, the human adipose stem cells were co-cultured with ADM sponges which promoted proliferation, tube formation, and migration of endothelial cells, and the GA-ADM group exhibited better migration behavior. There were no markable differences among expressions of pro-angiogenesis genes, including vascular endothelial growth factor, insulin-like growth factor-1, and epidermal growth factor. In a nude mouse model, the VT-ADM and GA-ADM pre-cultured with human adipose stem cells for 1 week in advance were implanted subcutaneously. The VT-ADM and the GA-ADM showed great histocompatibility without local redness, swelling, or necrosis. The vascular density of the local skin flap above the material was visualized using indocyanine green and showed no statistical difference between the two groups. The collagen tissue deposition in the pores and vessel formation within the sponges increased with time. Although VT-ADM had a higher degradation rate *in vivo*, the integrity of the two scaffolds was preserved. Collectively, the VT-ADM and the GA-ADM retained a natural matrix structure and presented biocompatibility. Thus, the above-mentioned two crosslinking methods for ADM sponges are safe and practicable. The novel ADM sponges with good physicochemical and biological properties are no longer limited to membrane tissue regeneration but could also realize structure remodeling where they act as scaffolds for a soft tissue filler and three-dimensional reconstruction of the tissue with strength requirements.

## Introduction

Porcine acellular dermal matrix (PADM) possesses advantages of being abundant in source, with high immune inertia and good mechanical strength (Janis et al., 2012). It is widely used in wound coverage, breast reconstruction, abdominal wall reconstruction, eyelid repair, stress urinary incontinence treatment, facial paralysis correction, and rhinoplasty (Guo et al., 2016; Bassetto et al., 2022). Reconstruction surgeries using PADM commonly achieve satisfactory results. Nowadays, the mainstream commercial PADM product is in a type of thin membrane, and common brands include STRATTICE, XenMatrix, and UniTrump. After removing antigens such as cells and glands, membrane-like PADM becomes a three-dimensional scaffold that retains a dermal matrix structure (Chen et al., 2022). Based on previous studies, PADM is mainly composed of type I and type IV collagen fibers, elastic fibers, and laminin, which is conducive to inducing epidermal cell crawling, fibroblast migration and endothelial progenitor cell aggregation (Lee et al., 2015). However, it is mostly derived from the dermal papillary layer with orderly collagen arrangement and dense tissue structure (Łabuś et al., 2021). Because the spatial structure is entirely preserved during acellular treatment, the vessel sprouting process is hindered to a certain extent. So far, the problem of vascularization delay after implantation remains unsolved, which increases the risks of postoperative reconstruction failure and graft necrosis.

In our previous studies (Zhu et al., 2019; 2020), we used laser drilling and freeze-thaw methods to increase the porosity of the PADM, which significantly improved its dense structure. However, free total porosity adjustment was a challenge. In addition, there were requirements for the thickness concerning specific clinical applications. For deep wounds or large soft tissue defects, overlying two or more pieces of PADM sheets may negatively impact timely vascularization and incorporation with host tissues after transplantation (Gowda et al., 2016), which would increase surgeons’ concern. Therefore, the requirements for flexible adjustments of PADM thickness and porosity are urgent in clinical practice.

Collagen is the main component of the extracellular matrix (ECM). A highly porous collagen sponge scaffold prepared by freeze-drying collagenous solution is a common biomaterial in tissue engineering. However, the mechanical strength of the non-crosslinking collagen sponge is weak (Sun et al., 2018). Thus, it is necessary to improve mechanical property and porosity of collagen sponges through crosslinking methods. To satisfy the reconstruction requirements of different tissues, the collagen sponge is further loaded with cells or coated on the surface for biomimetic simulation, but its microstructure is unable to completely mimic the natural ECM. During tissue regeneration, natural ECM plays a key role in undertaking signal and substance exchange between regenerative tissues and host tissues (Xue et al., 2022). Inspired by collagen sponges, we proposed an innovative concept of ADM sponges. The membrane-like PADM was first prepared into uniform microparticles that were smaller than 1 mm and retained dermal matrix natural microstructure. The microparticles were then crosslinked to form ADM sponges. The porosity of ADM sponges was controlled by the solid content of particle homogenate. ADM sponges have not been reported in published literature and are described in this article for the first time. In addition, the essential characteristics for biomaterials used in clinical practice should be good biocompatibility, adequate mechanical strength, appropriate degradation rate, and friend tissue integration.

Physical methods, such as ionizing irradiation, ultraviolet light treatment, dehydrothermal treatment, and chemical methods, such as aldehydes and isocyanates, are capable of introducing crosslinks to collagen materials (Sorushanova et al., 2019). In this study, vacuum thermal crosslinking was used to crosslink ADM microparticles. Belonging to physical techniques, the crosslinking is induced by temperature, and the resulting polymer is considered biocompatible and nontoxic. Exposed to high temperatures under a high vacuum, the free and bound water molecules were removed from collagen molecules (Weadock et al., 1995). Through condensation reactions, interchain crosslinked bonds were formed, including the amide bond formation and esterification reaction between carboxyl groups and hydroxyl groups (Gu et al., 2019). Without introducing crosslinking agents into the crosslinking structure, the thermal crosslinking product usually has inadequate mechanical strength, which requires an additional introduction of chemical agents (Zhou et al., 2021). Glutaraldehyde is a classic small molecular chemical crosslinking agent that has been shown to extensively stabilize collagen materials because of condensation reactions (Gu et al., 2019). The aldehyde groups of glutaraldehyde react with amino groups or hydroxyl groups in collagen fibers to form a stable crosslinked polymer. Glutaraldehyde is considered to be potentially cytotoxic caused by residual un-crosslinked agent which possibly forms a non-specific bond with the ECM. However, it has been reported that the low concentration of glutaraldehyde used to crosslink collagen scaffold could be fully removed after rinsing without cytotoxicity (Dorthé et al., 2022; Wu et al., 2022). Thus, to increase mechanical strength, the glutaraldehyde in a low concentration was introduced to ADM sponges after thermal crosslinking of matrix microparticles. Until now, there is no comparative study on physiochemical biological properties between thermal crosslinked and thermal-glutaraldehyde crosslinked ADM sponges. In addition, our previous research studies have shown that PADM co-cultured with human adipose stem cells (hADSCs) provided a pro-angiogenesis microenvironment and promoted the vessel formation in the early stage after implantation. We followed the co-culture scheme in this study, and pre-cultured ADM sponges with hADSCs for 1 week before subcutaneous transplantation.

**FIGURE 1:**
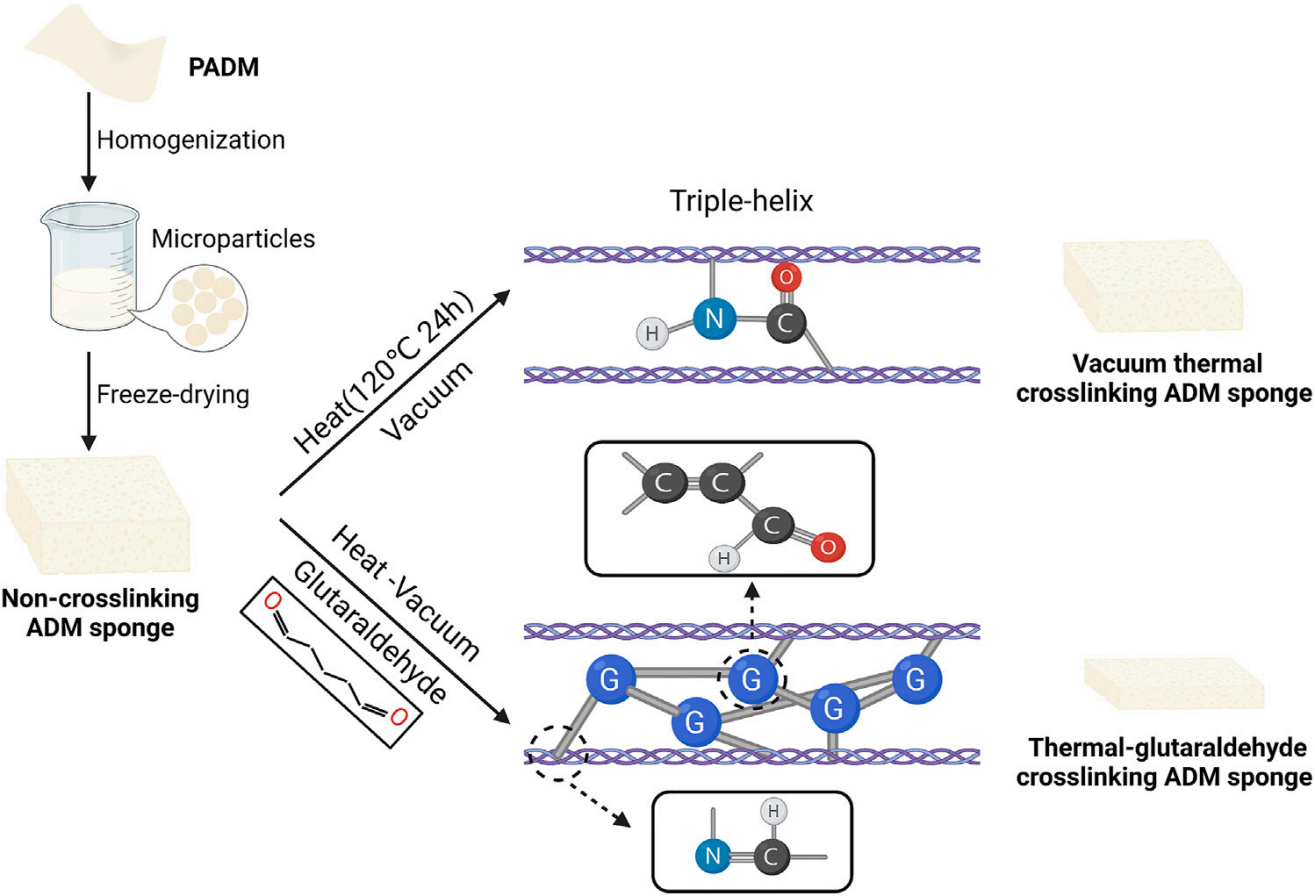
Process scheme of crosslinked ADM sponge preparation.

In the present study, we described two crosslinking methods to prepare ADM sponges from matrix microparticles, including thermal crosslinking and thermal-glutaraldehyde crosslinking (Figure 1). The structure and physicochemical properties of ADM sponges were evaluated. Then biocompatibility and angiogenesis of ADM sponges were explored *in vitro*. Finally, in a nude mice model, the ADM sponges were implanted subcutaneously on the back. The incorporation and remodeling process of ADM sponges, like material degradation, cell infiltration, collagen tissue deposition, and vessel ingrowth, were observed.

## Methods and Materials

### Material Preparation

The technique is provided by Jiangsu Unitrump Bio-medical Technology Co. (Jiangsu, China). The skin was obtained from healthy white pigs with an average weight of 50 kg. After carefully removing the epidermal layer and subcutaneous fat, the 0.5 mm split-thickness skin graft was manufactured. The sheet was completely submerged and sterilized by virus inactivation solution (self-prepared) mainly composed of peroxyacetic acid for 2 h. Then it was treated with a self-made mixed solution with 0.1–0.25% trypsin under the temperature of 30–40 °C for 30–60 min. The sheet was washed in purified water until the water was completely clear. The commercialized porcine acellular dermal matrix (PADM) was made, and the aforementioned procedure is protected by a patent (ZL201210060671.8).

Vacuum thermal crosslinking **(VT-ADM)**: with the homogenization treatment, the PADM composed of the basement membrane and dermal layer was shattered into particles of 1 mm in diameter. The tissue homogenates were frozen at -20 °C for shape formation and were freeze-dried. The sponge was crosslinked by heat (120 °C) for 24 h in a vacuum condition. The matrix **(VT-ADM)** was sterilized with epoxyethane with a concentration of 800 mg/L for 16 h and packaged until use.

Thermal-glutaraldehyde crosslinking **(GA-ADM)**: the sponge is crosslinked by the aforementioned dehydrothermal process and subsequent 0.1% glutaraldehyde treatment (room temperature, 24 h). The spongy material **(GA-ADM)** was thoroughly washed with purified water over 5 times until no free residual glutaraldehyde was detected. Then it was subjected to processes of freeze-drying, sterilization by 25 kGy gamma rays, and packaging for further use.

### Fourier-Transform Infrared (FT-IR) Spectral Measurements

The two-typed spongy ADM were characterized with an FT-IR (Nicolet Nexus 670 FTIR spectrometer) apparatus. The transmission mode was 4cm^−1^ intervals over the wavelength range of 4000-400cm^−1^ in a dry atmosphere at room temperature.

### Porosity

A certain amount of clean, dried spongy material was weighted (ms). A volumetric flask filled with ethanol was weighted m1. Then, the material was totally immersed in the flask, and the container was weighed together (m2). The fully saturated sample was carefully removed from the liquid, and the remaining ethanol and the pycnometer were weighted (m3). The porosity was calculated using the following equation:

$$Porosity=\frac{\left(m2-m3-ms\right)}{\left(m1-m3\right)}\times 100\%.$$

### Mechanical Properties, Water Contact Angle (WCA) Detection, and Differential Scanning Calorimetry (DSC)

Mechanical properties of the materials (*n* = 3) were measured using a mechanical analyzer (Model5542, Instron, United States). The non-crosslinked ADM sponge was 20 mm in length, 5 mm in width, and 4.3 mm in height. The VT-ADM was 20 mm in length, 5 mm in width, and 3.5 mm in height, while the GA-ADM was 20 mm in length, 5 mm in width, and 0.9 mm in height. The samples were stretched at a rate of 10 mm/min until fracture. The maximum load and Young’s modulus were further analyzed using Origin software (OriginLab, United States).

The WCA of non-crosslinked ADM sponge, VT-ADM, and GA-ADM (*n* = 3) was measured by using an OCA 20 contact angle system (Dataphysics, Germany) with a tilting base. The double-distilled water was dropped on the surface, and a video of the drop’s change over time was documented.

The thermal stability of the sample was characterized using Discovery DSC 250 (TA Instruments, United States). The heat flow difference among the non-crosslinking ADM sponge, VT-ADM, and GA-ADM during the process of temperature increase was detected. The samples were taken and sealed in a DSC crucible, and nitrogen was used as the protective gas. The temperature was in a range of 30–200°C with a heating rate of 10°C/min.

### Gross View and Scanning Electron Microscopy (SEM)

To observe the morphology of VT-ADM and GA-ADM, the gross features of the spongy materials were recorded with a single-lens reflex camera (Nikon, Japan). The surface morphology of the dried samples was scanned by SEM (ZEISS Gemini 300, Germany).

### Isolation, Culture, and Identification of hADSCs

hADSCs were isolated from five healthy female patients undergoing abdominal liposuction surgeries between November 2021 and March 2022. Written informed consent was provided by all patients. The average age of patients was 26 years (range, 24–28 years). The details of isolation, culture, and identification of hADSCs are shown in the Supplementary Material S1.

### Toxicity and Cytocompatibility of Spongy Materials

The VA-ADM and GA-ADM were entirely immersed in the DMEM containing 10% FBS and 1% streptomycin–penici1lin–amphotericin B solution (Gibco, United States) for 72 h, and the extracts were collected and passed through a 22-μm filter (Corning, United States). hADSCs (2000) were cultured in the complete DMEM and conditional extracts in a 96-well plate, and their proliferation were assessed on days 1, 3, and 5 with Cell Counting Kit (CCK)-8 (Dojindo, Japan). After incubation at 37°C for 2.5 h, the absorbance of samples was measured at a wavelength of 450 nm using an MP reader (Thermo, United States).

### hADSC Adhesion on the Surface of VT-ADM and GA-ADM

The viability of hADSCs (1×10^4^) seeded on VT-ADM and GA-ADM was measured with the Live/Dead cell viability assay (Yeasen Biotechnology, China) according to the manufacturer’s instruction on day 1 and day 3. The live cell (stained green) number was counted under per high-power field. Three fields were randomly selected from every sample and their average value was calculated.

The morphology of adhered cells was examined by SEM hADSCs (5 × 10^4^) were seeded on the circular spongy materials in size of the wells of a 24-well plate cultured for 3 days were examined by SEM. The samples were fixed overnight at 4°C in 2.5% glutaraldehyde and rinsed with phosphate-buffered saline (PBS) three times (15minutes each time). Then the samples were dehydrated through a graded series of ethanol (30-100%, V/V) and dried with a freezing-drier (YB-FD-1, SHYB Co., Ltd., China). Once dried, the samples were observed under an SEM (ZEISS Gemini 300, Germany). hADSCs (1×10^4^) seeded on VT-ADM and GA-ADM cultured for 2 days were stained by TRITC-tagged phalloidin according to the manufacturer’s instruction (Yeasen Biotechnology, China). The F-actin was specifically combined with phalloidin to exhibit cell morphology.

### Expression of Pro-Angiogenesis Genes

Gene expression related to angiogenic growth factors was evaluated by quantitative real-time PCR (RT-PCR). hADSCs (2×10^5^) were seeded into VT-ADM and GA-ADM in a 24-well plate and cultured for 48 h. Total RNA was extracted and reverse transcribed into cDNA with PrimeScript RT Master Mix using RNA purification kit and Reverse Transcription kit (with DNase) (EZBioscience, United States). Gene expression levels of vascular endothelial growth factor (VEGF), insulin-like growth factor 1 (IGF-1), and epidermal growth factor (EGF) were normalized to that of glyceraldehyde 3-phosphate dehydrogenase (GADPH) and quantified with the comparative Ct method. The primers synthesized by Sangon Biotech Co. (Shanghai, China) are mentioned in Table 1.

**TABLE 1:** Primer sequence for qPCR.

Cell	Gene	Forward primer	Reverse primer
hADSC	GAPDH	CAG​GAG​GCA​TTG​CTG​ATG​AT	GAAGGCTGGGGCTCATTT
	VEGF	ATC​GAG​TAC​ATC​TTC​AAG​CCA​T	GTG​AGG​TTT​GAT​CCG​CAT​AAT​C
	EGF	GAA​GCA​TTG​GAC​AAG​TAT​GCA​T	CAG​CTT​CTG​AGT​CCT​GTA​GTA​G
	IGF-1	AAA​AAT​CAG​CAG​TCT​TCC​AAC​C	CCT​GTG​GGC​TTG​TTG​AAA​TAA​A

### Human Umbilical Vein Endothelial Cell Proliferation and Tube Formation Assay

Human umbilical vein endothelial cells (HUVECs) were obtained from the cell bank of the Shanghai Institute of Cell Biology, Chinese Academy of Sciences (Shanghai, China). HUVECs were incubated in a low glucose medium (Gibco, United States) supplemented with 10% fetal bovine serum (CellMax, China) and 1% streptomycin–penici1lin–amphotericin B solution (Gibco, United States) and incubated at 37°C in a humidified atmosphere containing 5% CO_2_.

To collect the conditional medium (CM), the hADSCs (1×10^5^) seed on VT-ADM and GA-ADM were first cultured in complete DMEM for 24 h, washed three times with PBS, then cultured in serum-free DMEM for another 24 h. The supernatant was collected and centrifuged at 3,000 rpm to remove dead cells and debris. To improve the cell adhesion rate, the hADSCs were resuspended in a 100-μL complete culture medium, evenly instilled into the material, and cultured for 4 h before adding an abundant medium.

The proliferation of HUVECs was measured by using a Cell Counting Kit (CCK)-8 (Dojindo, Japan) after 1 and 3 days of culture. For the tube formation assay, HUVECs (1×10^4^/well) were seeded into a Matrigel-coated (Corning, United States) 96-well plate and treated with 50% CM and 50% serum-free endothelial culture medium, and those treated with serum-free endothelial culture medium were control groups. Then, the results were examined by bright-field microscopy at 4 h.

### Scratch Wound Healing Assay

HUVECs (2×10^5^/well) were seeded on a 6-well culture plate containing 3 ml complete medium/well in advance. When the confluence reached over 90%, a 200 μL pipette tip was used to generate scratch zones by streaking the well perpendicularly. The cells were washed with PBS two times, and the media were replaced with newly mixed media (50% CM and 50% serum-free endothelial culture medium) and serum-free endothelial culture medium for control. The scratch zones were observed after culture for 0, 6, and 24 h by light microscopy.

### *In Vivo* Experiment

All procedures were approved by the Ethics Committee of Shanghai Ninth People’s Hospital (assurance no. SH9H-2021-A091-SB). The hADSCs (2×10^5^) were pre-cultured with a slice of spongy material cut to 2 × 2 cm cross-sectional size (the height depending on the material itself) for 1 week before transplantation. Twenty-four 6-week-old male nude mice were randomly divided into two groups (two materials, and three timepoints, *n* = 4). After 1 week of acclimatization to the laboratory conditions, the mice were anesthetized by isoflurane inhalation (oxygen flowmeter of 400 ml/min), and their dorsal surfaces were disinfected. In the experiment group, a 2 × 2 cm rectangular-shaped skin flap on the back of mice was lifted, and the pre-treated material construct was implanted and fixed on the subcutaneous fascia. The incision was closed with an interrupted suture, and mice were allowed to recover from anesthesia. The observation endpoints were 2w, 4w, and 8w.

### Visualization of Flap Vascularization by Indocyanine Green Angiography

The mice were anesthetized using isoflurane inhalation (oxygen flowmeter of 400 ml/min). A 125-μL indocyanine green bolus (Dandong Yichuang Pharmaceutical Co., China) was injected into the tail vein of the mice (0.625 mg/ml and 0.3 ml/kg). Immediately after injection, the fluorescent detector (SPY imaging system; NOVADAQ Technologies Inc. Canada) was placed approximately 20 cm above the flap for video data acquisition. The perfusion zone on the local flap above the implanted material was white compared with the black non-perfusion filed.

### Macroscopic and Histological Analysis

At the ending timepoints, a rectangular skin flap was entirely removed along with the edge of the spongy material. Cutting the muscle layer beneath the material that obscured observation was avoided. The gross features of the material were examined and recorded with a single-lens reflex camera (Nikon, Japan). The materials and the surrounding skin were harvested and fixed in 4% paraformaldehyde at room temperature overnight. The samples were cut into sections, embedded in paraffin blocks, and cut into slices at a thickness of 5 μm.

To evaluate the cell infiltration depth, the sections were stained with hematoxylin and eosin (H and E).

To assess the newly born vessels, the CD31 immunohistochemical staining was used. The sections were incubated with anti-CD31 (Abcam, United States) at 1:500 dilution. The bound antibodies were 3,3′-diaminobenzidine (Beyotime Biotechnology, China), and the slices were counterstained with hematoxylin. The number of new blood vessels was calculated based on three random fields at ×200 magnification selected from each sample.

### Statistical Analysis

All quantitative results are presented as the mean ± standard error of the mean. Statistical comparisons were performed using Student’s t-test, one-way analysis of variance, or two-way analysis of variance, followed by Tukey’s *post hoc* test of data or Bonferroni’s method from three independent experiments. GraphPad Prism version 8.0 software (GraphPad, Inc. La Jolla, CA, United States) was used to analyze the data. Statistical significance was set at *p* < 0.05.

## Results and Discussion

### Analysis of Physicochemical Properties

**FIGURE 2:**
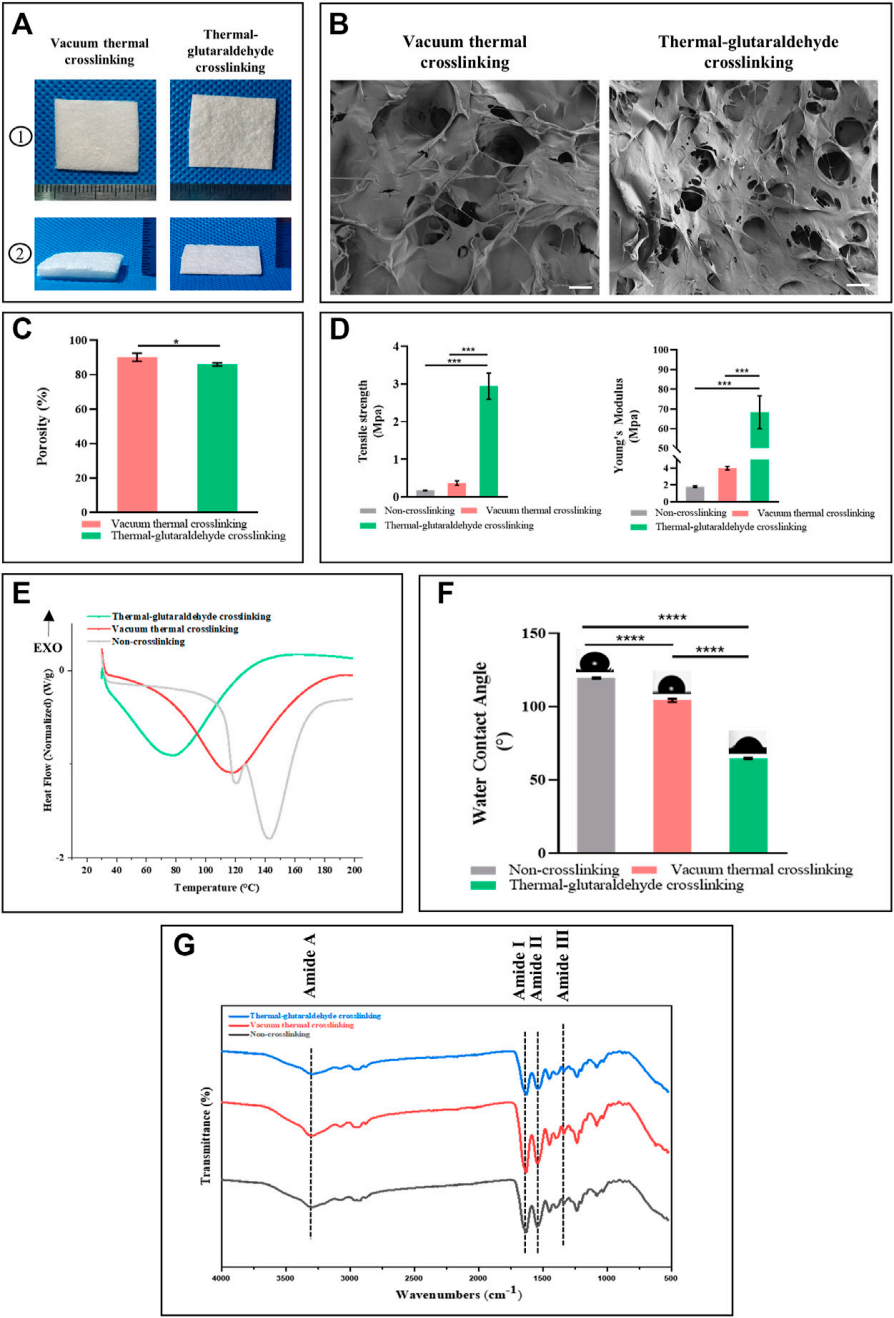
Physicochemical properties of the crosslinked ADM sponges. **(A)** Gross view of VT-ADM and GA-ADM. **(B)** scanning electron micrograph images of ADM sponges, bar = 200μm. **(C)** Porosity of ADM sponges (*n* = 3 for each group). **(D)** Mechanical properties including tensile strength and Young’s Modulus (*n* = 3 for each group). **(E)** Differential scanning calorimetry of ADM sponges (*n* = 3 for each group). **(F)** Water contact angle of ADM sponges (*n* = 3 for each group). **(G)** FT-IR spectra of ADM sponges. *p < 0.05, **p < 0.01, ***p < 0.001, and ****p < 0.0001.

The ADM sponges prepared by thermal crosslinking (VT-ADM) and thermal-glutaraldehyde crosslinking (GA-ADM) are shown in Figure 2A. Both VT-ADM and GA-ADM were porous sponges in off-white with a rough surface and nonspecific texture. The average thickness of VT-ADM was 3.5 mm, while that of GA-ADM was 0.9 mm. VT-ADM was significantly thicker than GA-ADM, indicating that the ADM sponge crosslinked by glutaraldehyde became denser with a decrease in void volume. However, both of them were thicker than membrane-like ADM with an average thickness of 0.33 mm (**A**-type product of the Unitrump), illustrating that ADM sponges were superior to the membrane-like ADM in the thickness. The surface morphology of VA-ADM and GA-ADM are displayed in Figure 2B. Pores of varied sizes were distributed irregularly on the surface, and the dermal matrix microparticles were arranged in the shape of flake.

During the freeze-drying process, the total porosity of ADM sponges was adjusted by controlling the solid content of ADM-microparticle homogenate, achieving the goal of free adjustment of porosity. To obtain a highly porous ADM sponge scaffold, the solid content should be controlled at 0.5–0.15% (this procedure is protected by an applying patent). The porosity of crosslinked ADM sponges was quantitatively analyzed using an ethanol immersion method (Figure 2C). The porosity of VT-ADM (mean 90.2%) was higher than the GA-ADM (mean 82.0%) with a statistical difference (*p*-value 0.0465). The result was consistent with the morphology observation. ADM sponge further crosslinked by the glutaraldehyde formed a firmer spongy structure connected by chemical covalent bonding (Guzelgulgen et al., 2021; Piola et al., 2022). Therefore, the pore interconnection inside the structure and total porosity decreased.

There was also a striking difference in mechanical strength between the VT-ADM and the GA-ADM (Figure 2D). No matter the tensile strength or Young’s modulus, the GA-ADM performed significantly better than the VT-ADM, indicating that the GA-ADM possessed a stronger anti-deformation ability. However, there was no marked difference between VT-ADM and non-crosslinking ADM sponge in mechanical property. The result revealed that the thermal crosslinking technique has little influence on mechanical properties, but the glutaraldehyde crosslinking technique could significantly improve the ADM sponge biomechanical property. Thermal crosslinking broke the original amide-water hydrogen bond among collagen fibers and newly formed a little stronger amide–amide hydrogen bond (Chen et al., 2020; Song and Wang, 2020). The glutaraldehyde formed covalent bonds with amino groups of collagen protein through Schiff base reaction or with hydroxyl groups of collagens and glycosaminoglycans to produce a more rigid molecular network, resulting in better mechanical strength of GA-ADM. (Islam et al., 2021; Shin et al., 2021). Additionally, the mechanical strength of the material increases with the porosity decrease (Wang et al., 2016; Wang et al., 2021; Zhang et al., 2022). Thus, the improvement of mechanical strength of GA-ADM partly contributed to porosity decrease.

The results of the ADM sponges before and after crosslinking using FIIR are shown in Figure 2G. The ADM sponges were made by PADM microparticles mainly composed of collagen. Collagen structure could be analyzed by using amide I (1,600–1800 cm^−1^), amide II (1,470–1,570 cm^−1^), amide III (1,250–1,350 cm^−1^), and amide A (3,300–3,500 cm^−1^) in the FIIR spectrum (Ji et al., 2020). The amide I at 1,630 cm^−1^ was from the C=O stretch vibration. The amide II reflected the cooperation between the bending vibration of N-H and stretching vibrations of C-N. The amide III at 1,335 cm^– 1^was mainly from N-H bending and C-N stretch vibration, while the amide A was mainly from the H-N and O-H vibrations (Wang et al., 2020). The amide I, amide II, and amide III reflect the characteristic structure (triple helix structure) of the collagen, while the Amide A reflects the hydrogen bond formation for the triple-helical structure stabilization (Zheng et al., 2021). The non-crosslinked ADM sponge, VT-ADM, and GA-ADM showed similar positions for absorbance peak (3,295 cm^**−1**^, 1,629 cm^**−1**^, 1,541 cm^**−1**^, and 1,335 cm^**−1**^) of classic amide bonds, corresponding to the amide A, the amide I, the amide II, and amide III, respectively. The amide I band moves to a lower region along with all characteristic peaks which weaken when the triple helix structure is destroyed (Zheng et al., 2021). There was no significant difference in the amide band absorption peak among the three ADM sponges, implying that the triple helix structure was almost unchanged after crosslinking. All in all, both vacuum thermal crosslinking and glutaraldehyde crosslinking have little influence on the natural structure of collagen and maintain biofunction of the complex and comprehensive dermal matrix.

The thermal behavior among the non-crosslinking ADM sponge, VT-ADM, and GA-ADM were analyzed using DSC. The thermal property of collagen materials was characterized by its denaturation temperature and enthalpy (da Silva Brum et al., 2021). Three groups had different denaturation temperatures and enthalpies (Figure 2E). The denaturation temperatures of non-crosslinked ADM sponge, VT-ADM, and GA-ADM were 142.72°, 118.07°, and 78.62°, respectively, while the corresponding enthalpy values were 320.49J/g, 374.26 J/g, and 335.19 J/g. The denaturation temperature represented the transition of the collagen triple helix to a random coil, while the melting enthalpy indicated the heat required by helix-coil transformation (Chen et al., 2019; Park et al., 2020). In the actual clinical setting, the intended objects for application are warm-blooded humans (37°). Thus, the three types of ADM sponges are able to maintain thermal stability and the original structure in the humoral environment. The DSC results proved that both the VT-ADM and the GA-ADM had stable thermal properties in clinical practice.

The water contact angle reflects the hydrophilicity of ADM sponges. 0–90° is the hydrophilic contact angle, while 90–180° is the hydrophobic contact. The water contact angles of VT-ADM and GA-ADM decreased significantly compared with the non-crosslinking ADM sponge (Figure 2F), illustrating the hydrophilicity of the crosslinking ADM sponges increased. The average contact angles of non-crosslink ADM sponge, VT-ADM, and GA-ADM were 119.4°, 104.27°, and 67°, respectively. The hydrophilicity of the GA-ADM was better than that of the VT-ADM due to more hydrophilic groups. The hydrophilic groups, such as -COH, -OH, and -COOR, existing in the glutaraldehyde formed hydrogen bonds with water molecules, resulting in the improvement of hydrophilicity of the GA-ADM (Sung et al., 2000). The scaffold with a hydrophilic surface is conducive to cell adhesion and proliferation (Sang et al., 2018). The GA-ADM with a hydrophilic surface could be expected to be more biocompatible (Wang et al., 2018).

In short, the VT-ADM prepared by thermal crosslinking and the GA-ADM prepared by thermal-glutaraldehyde crosslinking are porous scaffolds with a certain thickness. Both of them preserve the spatial structure of collagen and the natural microstructure of dermal matrix, and exhibit good thermal stability for clinical applications. To quantify the degree of crosslinking, the mechanical properties and water contact of non-crosslinking ADM sponge, VT-ADM, and GA-ADM were measured (Table 2) **(** Koosha et al., 2019). In addition, the existence and function of other components of the dermal matrix, such as elastin, hyaluronic acid, and matricellular proteins, in ADM sponges rather than collagen would be explored in further studies (Huang et al., 2022).

**TABLE 2:** Tensile strength, Young’s Modulus, and contact angle of ADM sponges.

Sample	Non-crosslinking sponge	VT-ADM	GA-ADM
**Tensile strength (Mpa)**	**0.173 ± 0.012**	**0.367 ± 0.100**	**2.940 ± 0.603**
**Young’s Modulus (Mpa)**	**1.800 ± 0.165**	**3.993 ± 0.367**	**68.360 ± 14.420**
**Contact angle (°)**	**119.400 ± 0.702**	**104.300 ± 1.823**	**64.67 ± 0.896**

The values are presented as the mean ± standard error of the mean.

Compared with the VT-ADM, the GA-ADM has increased crosslinking degree, denser structure, higher hydrophilicity, and stronger mechanical strength, but less total porosity. It was our first try to adjust the thickness of ADM in a type of sponge, which broke through the limitation of the inherent porcine dermal tissue thickness (Adelman et al., 2014; Aragoneses et al., 2021). However, the thickness of ADM sponges cannot be freely modified for personalized customization. The preparing technique needs to be further optimized to satisfy different requirements in thickness.

### Cytocompatibility of ADM Sponges

**FIGURE 3:**
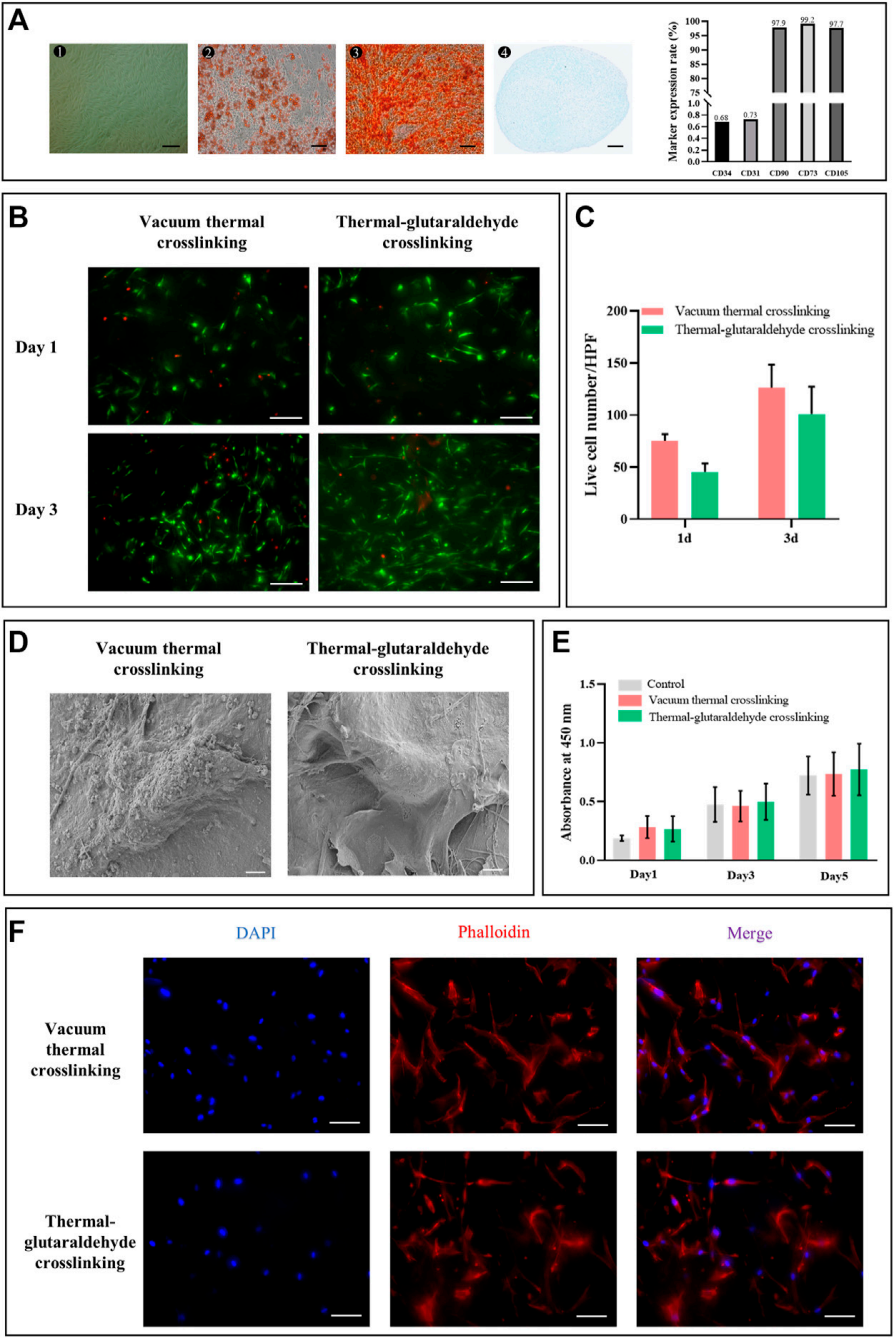
Biocompatibility of the crosslinked sponges. **(A)**Morphology (bar = 200 μm) and differentiation potential (bar = 100 μm) of hADSCs. Marker expression rate was analyzed by flow cytometry. **(B)** Live and dead staining of hADSCs on the ADM sponges crosslinked by the vacuum thermal method or thermal-glutaraldehyde method, bar = 100 μm. **(C)** Calculation of live cells per high power field on the crosslinked ADM sponges on days 1 and 3. (n = 3 for each group). **(D)** SEM images of hADSCs on the surface of ADM sponges, bar = 5 μm. **(E)** hADSC proliferation on days 1, 3 and 5 cultured in the extracts from VT-ADM, GA-ADM, and complete DMEM, respectively (*n* = 3 for each group). **(F)** hADSC adhesion to AT-ADM and GA-AMD stained by phalloidin, bar = 100 μm (blue for nucleus; red for cytoskeleton). *p < 0.05, **p < 0.01, ***p < 0.001, and ****p < 0.0001.

The human adipose stem cells (hADSCs) used in this study were identified (Figures 3A). The surface markers of hADSCs (P3) were analyzed by flow cytometry. CD105 (97.7%), CD75 (99.2%), and CD90 (97.9%) were of high expression, while CD34 (0.68%) and CD31 (0.73%) were of low expression. CD31 was the marker of the endothelial cells, and CD34 was the marker of hematopoietic progenitor cells. The low expression of CD31 and CD34 indicated that the hADSCs after proliferation still kept high purity (the details are shown in the Supplementary Figure S1). The tri-differentiation of the hADSCs was further verified (Dominici et al., 2006). hADSCs grew adherently with a typical spindle shape, which induced adipogenesis (lipid droplets were stained red by Oil red), osteogenesis (calcium nodus were stained red by Alizarin red), and chondrogenesis (cartilage ball was stained blue by Alcian blue). Therefore, hADSCs extracted from adipose tissues had high purity and stemness.

hADSCs are common seed cells for tissue engineering scaffolds due to their advantages of multiple differentiation, cytokine secretion, and rich source. Kim SH et al. found that the **poly(** **l** -lactide-co-caprolactone) scaffold containing hADSCs promoted angiogenesis *in vivo* (Kim et al., 2021). Bi H et al. also reported that the hADSCs facilitated the tuber formation of endotheliocytes (Bi et al., 2019). Thus, we followed the co-culture scheme in this study to pre-culture ADM sponges with hADSCs. The treatment had been proven to effectively promote vascularization in the early phase after membrane-like PADM implantation through the angiogenic microenvironment. Before constructing the co-culture system, we explored the cytocompatibility of VT-ADM and GA-ADM.

The hADSCs adhesion morphology on the surface of VT-ADM and GA-ADM was observed by SEM. Those hADSCs adhering to the ADM sponges were spindle-like and extended pseudopodia (Figures 3D). The adhesion morphology of hADSCs was also observed using cytoskeleton staining. The nucleus was stained blue, while the cytoskeleton was stained red. The hADSCs adhering on the surface of VT-ADM and GA-ADM exhibited a spindle shape, and their pseudopod extended toward the poles (Figures 3F). The proliferation of hADSCs was observed by Live/Dead staining. The live cells stained green distributed unevenly on the surface of the ADM sponges on day 1, and their numbers significantly increased on 3 days, showing the pattern of “overall distribution, local aggregation” (Figure 3B). The living hADSCs on days 1 and 3 were further counted under a high-power field, and no statistical difference was found between the two groups (Figure 3C). The cytotoxicity of VT-ADM and GA-ADM was researched indirectly (Figure 3E). Setting the complete medium as control, the influence of extracts of VT-ADM and GA-ADM on cell proliferation was evaluated. There was no significant difference (*p*-value > 0.5) among groups using CCK8-kit, indicating that the material extract had little influence on hADSC proliferation. The results proved the effectiveness of sterilization methods which could be considered that ADM sponges had no cytotoxicity.

All in all, both VT-ADM and GA-ADM had no cytotoxicity and were suitable for cell proliferation and adhesion without significant difference. The ADM sponge crosslinked by the glutaraldehyde in low concentration not only improved the stability of crosslinking but also had little cytotoxicity.

### Angiogenesis of hADSC-Sponge Co-culture System

**FIGURE 4:**
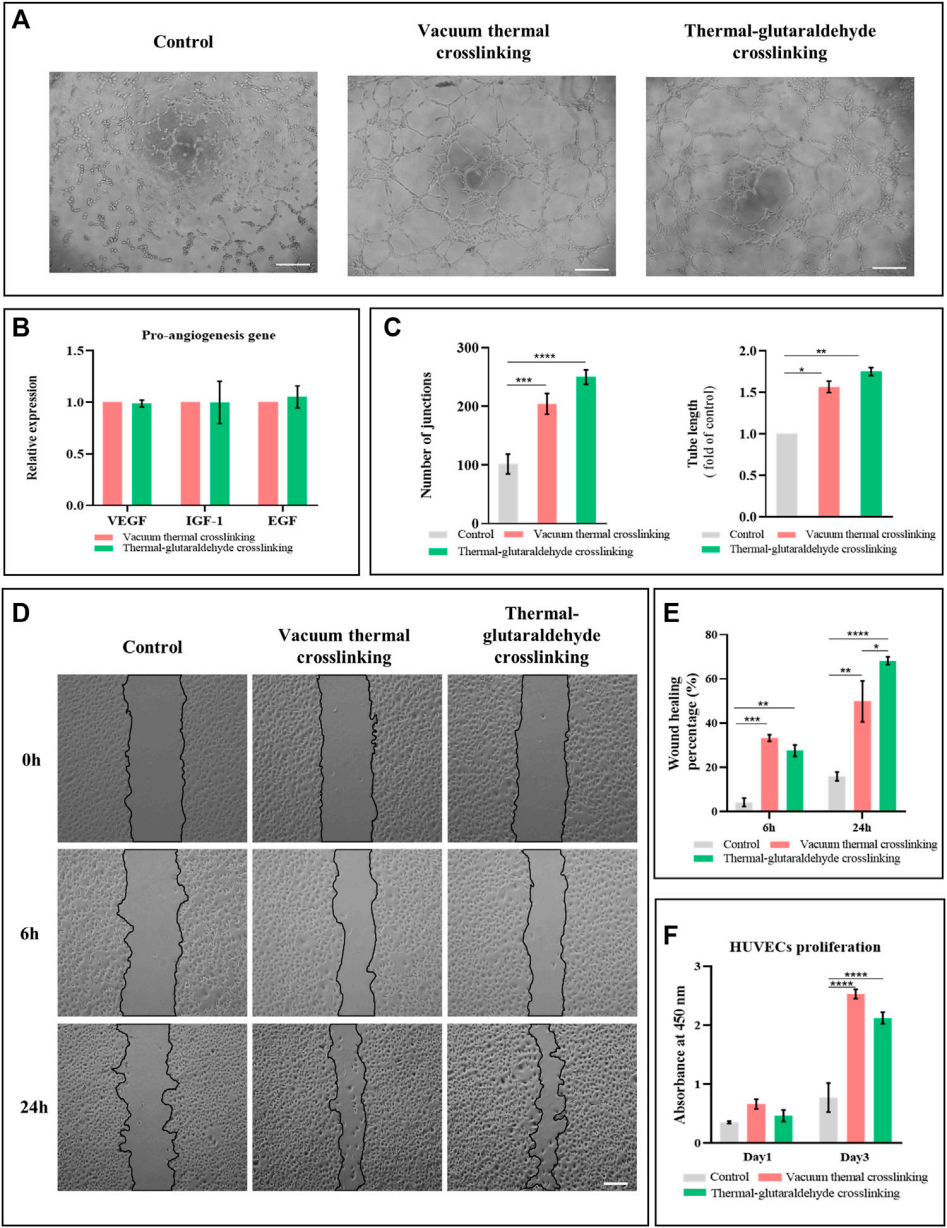
Pro-vascular ability of VT-ADM and GA-ADM *in vitro*. **(A)** Tube formation induced by conditional medium and blank medium at 4 h, bar = 200 μm. **(B)** Pro-angiogenesis gene expression levels of VT-ADM and GA-ADM (*n* = 3 for each group). **(C)** Statistical analysis of the number of junctions and total tube length of the tube formation at 4 h (*n* = 3 for each group). **(D)** Wound healing test induced by conditional medium and blank medium at 0, 6, and 24 h, bar = 200 μm. **(E)** Wound healing percentage at 6 and 24 h in three groups (control, VT-ADM, and GA-ATM groups, *n* = 3 for each group). **(F)** HUVEC proliferation cultured in conditional media and blank medium on days 1 and 3 (*n* = 3 for each group). *p < 0.05, **p < 0.01, ***p < 0.001, and ****p < 0.0001.

The angiogenesis of VT-ADM or GA-ADM co-cultured with hADSCs were compared through a series of *in vitro* experiments. The conditional medium was the supernatant of the ADM sponge co-cultured with hADSCs in a serum-free endothelial medium for 2 days. The tube formation on the matrix gel induced by the conditional medium or basic medium (serum-free endothelial medium) for 4 h is shown in Figure 4A. The control group (basic medium) has scattered junctions and branches without a classic circle structure. Increased branches and circle structure were observed in the experimental groups. The junctions and total branching length were calculated using ImageJ software. Both the VT-ADM group and GA-ADM group were larger than the control group, but there was no statistical difference between them.

In terms of angiogenesis, VEGF induces vessel sprouting and endothelial cell proliferation, promotes angiogenic response, and regulates vasodilatation (Ferrara, 1999); IGF-1 contributes positively by exerting the anti-apoptosis effect, strengthening the metabolization of endothelial cells, and promoting the migration of vascular smooth muscle cells (Lin et al., 2017); EGF plays a role in regulating proliferation and migration of endothelial cells (Boonstra et al., 1995). It was found that the pro-angiogenesis gene expressions of VEGF, IGF-1, and EGF of hADSCs adhering and proliferating on the VT-ADM and the GA-ADM had no difference (Figure 4B). This suggests that the hADSCs-ADM sponge co-culture system secreted pro-angiogenic cytokines, but the VT-ADM group and GA-ADM group had no marked difference in the gene expression of VEGF, IGF-1, and EGF.

Endothelial cells migrate during vasculogenesis and angiogenesis depending on microenvironments and varied signals (Michaelis, 2014). The effect of ADM sponge-hADSCs co-culture system on the migration of HUVECs was studied by the scratch wound healing assay. Three groups were the control group (serum-free medium), the VT-ADM group, and the GA-ADM group (the supernatant of the co-culture system). The creeping behaviors of HUVECs at 0, 6, and 24 h among three groups are displayed in Figure 4D. HUVECs climbed from the border of the gap and migrated to the middle of the gap with time. The HUVECs of the VT-ADM group and the GA-ADM group moved faster than the control group. The wound healing rates (healing area/original gap area) at 6 and 24 h were calculated. At 6 h, the wound healing rates of the GA-ADM group and the VT-ADM group were higher than the control group, but there was no obvious difference. At 24 h, the wound healing rate of the GA-ADM group (mean: 68.2%) was significantly larger than that of the VT-ADM group (mean: 49.8%). The endothelial cell migration is promoted by the growth factors like basic fibroblast growth factor when the endothelial monolayer is damaged. Upon the growth factors stimulation, the cells at the wound boundary exhibit directed migration toward the gap in the sheet. This indicates that the GA-ADM co-cultured with hADSCs was conducive to forming a suitable microenvironment for HUVEC migration.

The influence of the VT-ADM group and the GA-ADM group (the supernatant of the co-culture system) on proliferation of HUVECs was evaluated (Figure 4F). The serum-free medium was the control group. The aforementioned three groups yielded no significant difference in OD values on day 1, indicating that little influence on proliferation was observed. The OD values of the three groups were higher on day 3 than those on day 1. Furthermore, the OD values of the VT-ADM group and the GA-ADM group were larger than that of the control group, indicating that the ADM sponge-hADSCs co-culture system facilitated the proliferation of HUVECs. However, there was no significant difference between the VT-ADM group and the GA-ADM group on days 1 and 3, indicating that no noticeable difference existed in promoting HUVEC proliferation.

In summary, the pro-angiogenesis of the VT-ADM and the GA-ADM co-cultured with hADSCs were compared from proliferation, migration, and tube formation of HUVECs. Both the co-culture systems improved the proliferation, migration, and tube formation, and the GA-ADM system has an obvious advantage in promoting migration.

### *In Vivo* Experiments

**FIGURE 5:**
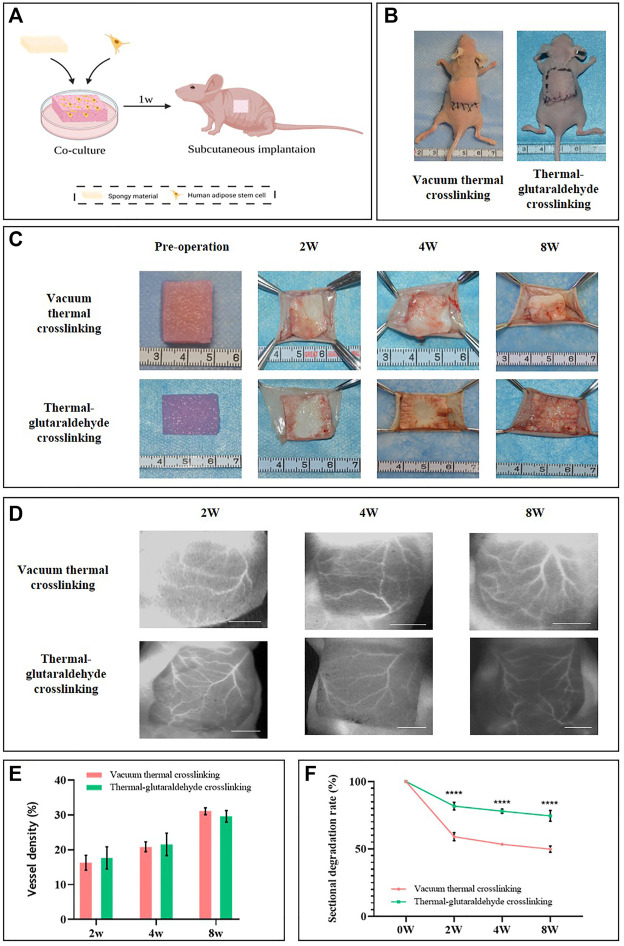
*In vivo* experiments. **(A)** Schematic diagram of animal experiments was created by BioRender.com. **(B)** Intra-operative views after VT-ADM and GA-ADM implantation. **(C)** Gross views of the ADM sponges before operation and at observation ending timepoints. **(D)** ICG images of blood vessels on local skin flap anterior to VT-ADM and GA-ADM, bar = 5 mm. **(E)** Vessel density of local skin flaps at 2w, 4w, and 8w was analyzed by the AngioTool (*n* = 3 for each group). **(F)** Degradation rates of VT-ADM and GA-ADM at 2w, 4w, and 8w (*n* = 3 for each group). *p < 0.05, **p < 0.01, ***p < 0.001, and ****p < 0.0001.

VT-ADM and GA-ADM were pre-cultured with hADSCs *in vitro* for 1w in advance and then were implanted subcutaneously on the back of nude mice. The material degradation and vascularization, cell infiltration, and extracellular matrix deposition were observed at 2w, 4w, and 8w. The schematic diagram of the animal experiment is shown in Figure 5A. The images immediately after ADM sponge implantation are displayed (Figure 5B). VT-ADM and GA-ADM were completely embedded in the central area on the back, and the incisions were closed using interrupted sutures. No wound disruption or local redness and swelling were observed during the follow-up.

The degradation of ADM sponges *in vivo* is shown (Figure 5C). The VT-ADM and the GA-ADM were cut into cubes with a cross-section in size of about 2 × 2 cm (the thickness was decided by the material itself). Following the increase in time, the VT-ADM and the GA-ADM experienced degradation from the edge to the middle. For the VT-ADM in particular, its edge became irregular. Near the dorsal tissues, the vessels grew from the periphery to the center. At 2 weeks, the vascular distribution mainly concentrated on the peripheral zone of the sponges. At 8 weeks, the vessels grew into the central zone where no necrosis occurred. This suggests that although the thickness increased significantly, ADM sponges could realize complete vascularization that are safe enough for clinical applications. The size-changing of cross-sections was counted (Figure 5F). It was found that the degradation of the VT-ADM was obviously faster than the GA-ADM. Because glutaraldehyde interacts with amine or hydroxyl groups, interfibrillar and intrafibrillar bonds increases, and the *in vivo* degradation time increases as well (Doustdar et al., 2022). Rapid degradation of the ADM provides an unsuitable microstructure for collagen deposition and neovascularization (Lee et al., 2015). Therefore, delaying the degradation process to some extent is conducive to tissue regeneration.

The newly grown vessels of ADM sponges originated from the local skin flap above the material or the dorsal muscular layer below the material. The growth pattern of the dorsal side of ADM sponges was described as mentioned earlier. Indocyanine green (ICG) angiography has been widely used in assessing skin flap perfusion (Han et al., 2022). Therefore, the ICG method was used to visualize the vascular distribution of the local skin flap (Figure 5D). The black area was where the sponge was located, while the white indicated the blood vessels. At 2w, the side branches dominated with few main branches in the flap. At 8w, the main branches increased while the side branches communicated mutually. It may relate to the angiogenic stimulated niches for skin flaps provided by implanted ADM sponges (Wu et al., 2021). The increasing vessels of the flap in turn promoted the vascularization of the ADM sponges. Additionally, the vascular distribution also followed the pattern of filling the periphery before its growth toward the center. The vascular density of the local flap was calculated by AngioTool software (Figure 5E) and was found to increase with time, which was consistent with the observation results of the adverse side of the biomaterial. However, there was no obvious difference between the VT-ADM group and the GA-ADM group at 2w, 4w, and 8w. Thus, the VT-ADM and the GA-ADM promoted vessel growth of local flap without significant difference.

In conclusion, VT-ADM and the GA-ADM could realize adequate vascularization *in vivo* without material necrosis. The degradation speed of the GA-ADM was slower than the VT-ADM. Therefore, the GA-ADM provided a more durable scaffold to induce new tissue formation.

### Histological Staining Observation and Analysis

**FIGURE 6:**
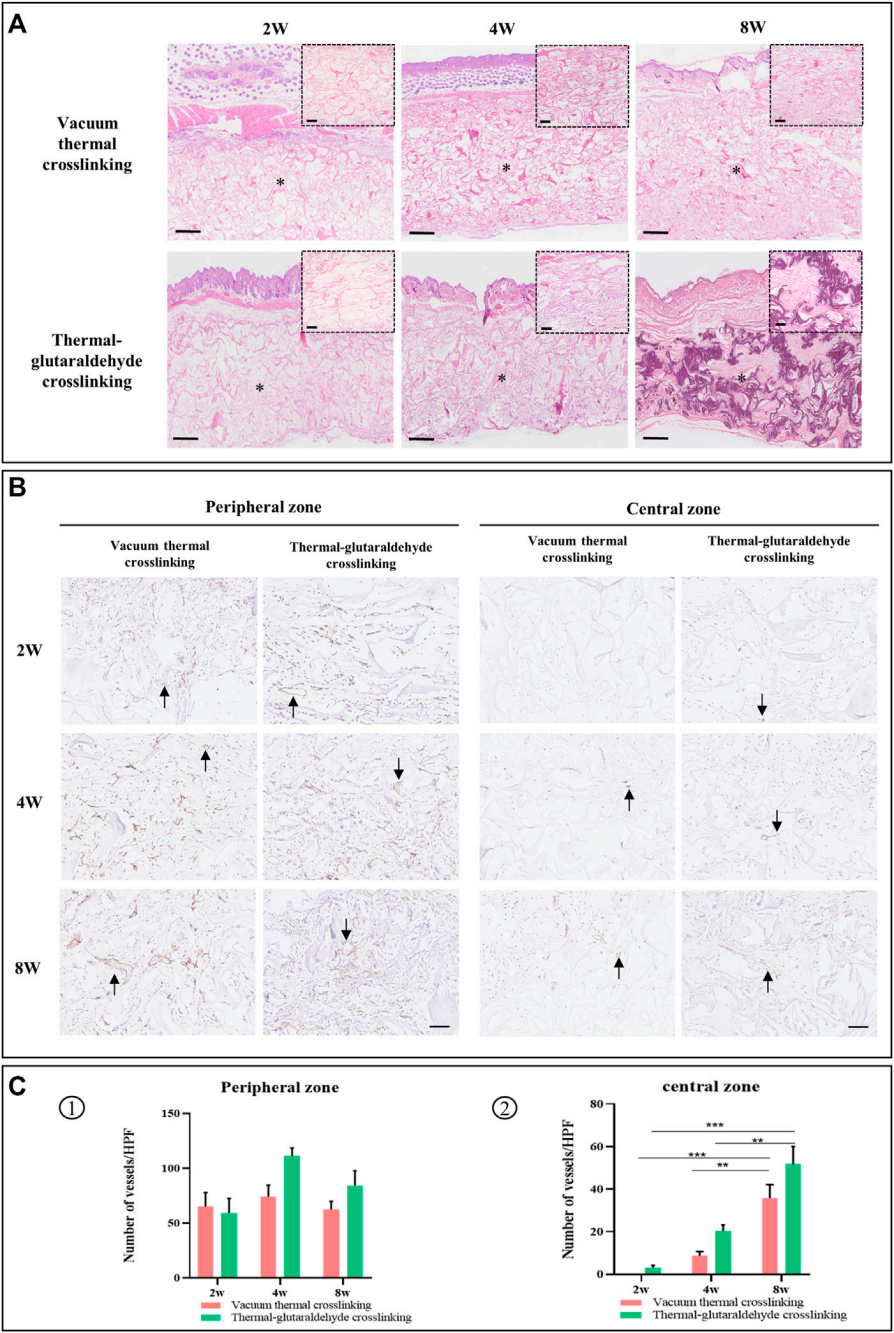
Histopathological observation. **(A)** HE staining. The small picture in the top corner is the local enlarged view to display clearly. Bar in the big picture = 500μm, bar in the small picture = 100 μm (the M character indicates material). **(B)** Immunohistochemical staining for CD31 in materials, and black arrow points new vessels, bar = 100 μm. **(C)** Statistical analysis on the number of vessels in the peripheral zone and the central zone at 2w, 4w, and 8w (*n* = 3 for each group). *p < 0.05, **p < 0.01, ***p < 0.001, and ****p < 0.0001.

To observe the cell infiltration, fibrous tissue formation and blood vessel growth, the tissue samples were stained with HE staining and immunohistochemical staining (CD31, the specific marker of the endothelial cells). The vertical section of the ADM sponge was exhibited using HE staining (Figure 6A). The fibrous tissues were formed to connect the skin and the sponge marked by an M character. The images on the upper right amplified the part of the sponge. As a highly porous scaffold, the ADM sponges were infiltrated by cells from the upper and lower edge meeting the host tissues gradually to the central zone. The whole layer of the GA-ADM was infiltrated at 4w, but the part of the central zone of VT-ADM did not see infiltration even at 8w. In addition, the collagen tissue deposited in the porous structure and increased following the duration in time (Wang et al., 2015). At 8w, the porous architecture of GA-ADM was completely replaced by the collagen tissue, but those in VT-ADM remained blank in the central zone. The results demonstrated an adequate extracellular matrix deposition which was mainly composed of collagen tissues. The result of extracellular matrix deposition was consistent with host cell infiltration, indicating that the GA-ADM had faster incorporation with the surrounding tissues than the VT-ADM (Li et al., 2015). The VT-ADM was significantly thicker than the GA-ADM, so the remodeling process on the central zone of the VT-ADM was inferior to the GA-ADM. At 2w, 4w, and 8w, the scaffold structure of the VT-ADM and the GA-ADM could be observed, illustrating that the VT-ADM and the GA-ADM provide stable scaffolds for tissue regeneration.

The vascularization of the ADM sponge was exhibited by immunohistochemical staining. The new vessels were stained in brown and indicated by arrows. The vessels were observed both in the peripheral area and the central area (Lai et al., 2020). In particular, in the central zone, the number of vessels increased as time went on. In terms of the peripheral area, the VT-ADM and the GA-ADM were mainly occupied by microvessels at 2w, and the diameters of some vessels significantly increased at 8w. Counting the vessel numbers in the surrounding area, it has been found that there was no statistical difference in the vascular numbers between two types of the ADM sponges at varied timepoints, or on the number of vessels using a self-calibrated method, respectively. This suggests that for the VT-ADM and the GA-ADM, vascular ingrowth occurred first, followed by an adaptive remodeling process. In terms of the central area, few vessels were observed in the GA-ADM, but no vessel was in the VT-ADM due to its thickness at 2w. Then the vascularization of the two groups was improved at 4w. The numbers of vessels of VT-ADM and GA-ADM were greater at 8w than those at 4w. However, there was no statistical difference in the number of vessels in the central region between the aforementioned two groups at 2w, 4w, and 8w. Angiogenesis was primarily regulated by pro-vascular cytokines secreted by the adhering cells in the material (Barnhill and Wolf, 1987; Phillippi, 2022). The results reflected that though the VT-ADM was thicker than the GA-ADM, the vascularization of the implanted VT-ADM was unaffected.

To sum up, both VT-ADM and GA-ADM effectively induced cell infiltration, vessel ingrowth, extracellular matrix deposition, and provided a long-term stable matrix scaffold to receive a similar mechanical strength and biological function to those of original tissues. They were fully vascularized *in vivo* and exhibited a difference in tissue integration due to material thickness. Combined with the physicochemical properties and biological performance, the thicker VT-ADM in 3.5 mm depth with weaker mechanical strength is suitable for soft tissue augmentation, such as deep wound coverage, breast augmentation, and buccal region depression filler; the thinner GA-ADM in 0.9 mm depth with stronger mechanical strength and slower degradation was appropriate for tissue substitute with strength requirement, including lower eyelid retraction repair, tarsal plate defect reconstruction, and eyeball wall damage repair.

## Conclusion

The ADM sponge prepared by the ADM microparticles is a highly porous scaffold that preserves the dermal matrix’s natural microenvironment. The VT-ADM produced by thermal crosslinking under a high vacuum is thicker with weaker mechanical strength, while the GA-ADM made by thermal-glutaraldehyde crosslinking is thinner with stronger mechanical property. Both of them have good biocompatibility without cytotoxicity and are considered novel biofriendly ADM materials. The VT-ADM and the GA-ADM could induce fast cell infiltration, adequate extracellular matrix deposition, and sufficient vascularization as great regenerative biomaterials. Thus, the crosslinked ADM sponges provide the possibility to repair complex wounds of different depths, and decrease the risks of infections and unnecessary gaps between the material and host tissues caused by overlying several membrane-like ADM sheets. Collectively, the two types of ADM sponges are safe and reliable novel biomaterials for future clinical applications.

## Data Availability

The original contributions presented in the study are included in the article/Supplementary Material; further inquiries can be directed to the corresponding authors.
