# Supplementary material for: A Comparative Study on Two Types of Porcine Acellular Dermal Matrix Sponges Prepared by Thermal Crosslinking and Thermal-Glutaraldehyde Crosslinking Matrix Microparticles
Source: Front Bioeng Biotechnol. 2022 Aug 5;10:938798. doi: 10.3389/fbioe.2022.938798 (PMC9388789; doi:10.3389/fbioe.2022.938798)
Supplement: Supplementary file 1 [file DataSheet1.docx]

Supplementary Material

**Isolation, culture and identification of hADSCs**

hADSCs were isolated from 5 healthy female patients undergoing abdominal liposuction surgeries between November 2021 to March 2022. Written informed consent was provided by all patients. The average age of patients was 26 years (range, 24-28years). The adipose tissue was first sterilized by being immersed in 3% streptomycin-penici1lin-amphotericin B solution (Gibco, USA). An equal volume of 0.15% (W/V) collagenase NB4 (Serva, Heidelberg, Germany) in serum-free low-glucose Dulbecco's Modified Eagle's Medium (DMEM, Gibco, USA) was added to washed adipose tissue, and the mixture was digested at 37℃ for 2h to a homogeneous state. The lipoaspirate was centrifuged at 1200 rpm for 8 minutes at 37℃. The cells were concentrated and then resuspended in DMEM containing 10% fetal bovine serum (CellMax, China) and 1% streptomycin-penici1lin-amphotericin B solution (Gibco, USA) and seeded into 10mm culture dishes. The cells were incubated at 37℃ contained 5% CO_2_ and were cultured to 90% confluence before passaging. hADSCs of Passages 3 to 4 were used for the experiments.

hADSCs were tested for their ability to undergo tri-lineage differentiation into adipocytes, osteoblasts, and chondrocytes. hADSCs (Passage 3) at a density of 2×10^5^ cells/mL were seeded into six-well plates, induced for 2 weeks with adipogenic (Cyagen, China) differentiation media, 3 weeks with osteogenic (Cyagen, China) differentiation media, and 4 weeks with chondrogenic (Cyagen, China) differentiation media according to manufactures' instructions. The cells were then fixed in 4% paraformaldehyde and stained with either Oil Red O (Beyotime, China), Alizarin red (Beyotime, China), or Alcian blue (Beyotime, China) according to standard procedures.

The surface markers of hADSCs were detected by flow cytometry. hADSCs (Passage 3) were harvested and incubated with fluorescein isothiocyanate or phycoerythrin-conjugated antibodies against CD73, CD90, CD105, CD31 and CD34 (Santa Cruz Biotechnology, Inc., Santa Cruz, CA, USA) at 37°C for 30 min in the dark, washed, and resuspended in PBS and detected by flow cytometry (BD Biosciences, San Jose, CA, USA).

**Figure**


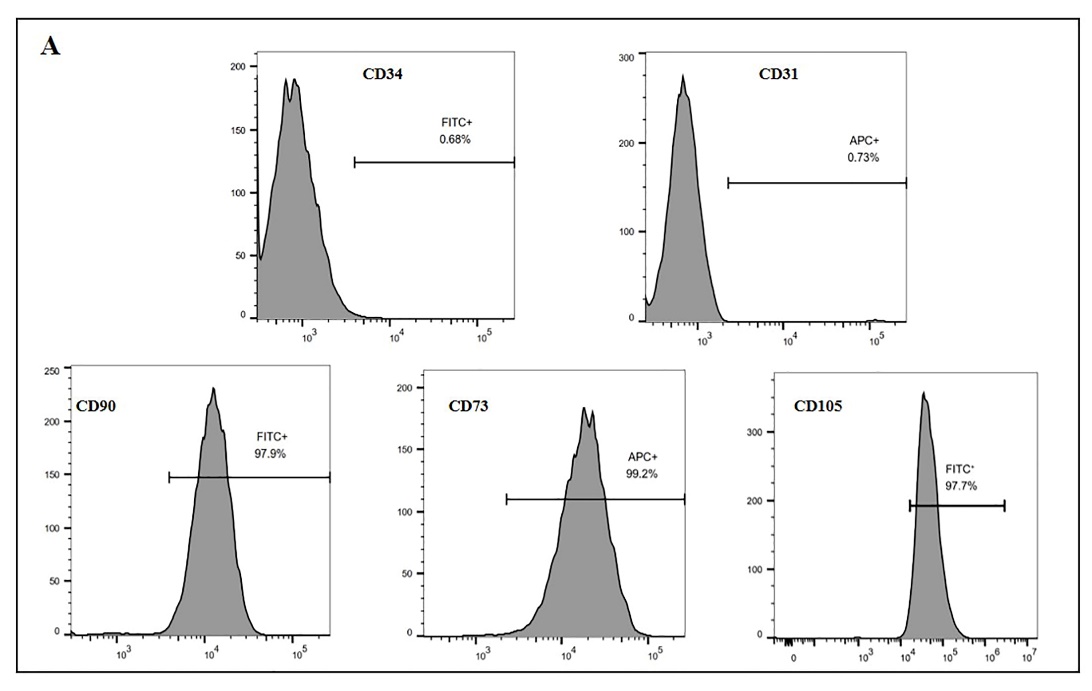


Supplementary Figure 1 The results of hADSCs markers analyzed by flow cytometry.
